# Different Grp94 components interact transiently with the myocilin olfactomedin domain *in vitro* to enhance or retard its amyloid aggregation

**DOI:** 10.1038/s41598-019-48751-8

**Published:** 2019-09-04

**Authors:** Dustin J. E. Huard, Alex P. Jonke, Matthew P. Torres, Raquel L. Lieberman

**Affiliations:** 1 School of Chemistry & Biochemistry, Georgia Institute of Technology, Atlanta, GA 30332 USA; 2 School of Biology, Georgia Institute of Technology, Atlanta, GA 30332 USA

**Keywords:** Chaperones, Protein aggregation

## Abstract

The inherited form of open angle glaucoma arises due to a toxic gain-of-function intracellular misfolding event involving a mutated myocilin olfactomedin domain (OLF). Mutant myocilin is recognized by the endoplasmic reticulum (ER)-resident heat shock protein 90 paralog, glucose regulated protein 94 (Grp94), but their co-aggregation precludes mutant myocilin clearance by ER-associated degradation. When the Grp94-mutant myocilin interaction is abrogated by inhibitors or siRNA, mutant myocilin is efficiently degraded. Here we dissected Grp94 into component domains (N, NM, MC) to better understand the molecular factors governing its interaction with OLF. We show that the Grp94 N-terminal nucleotide-binding N domain is responsible for accelerating OLF aggregation *in vitro*. Upon inhibiting the isolated N domain pharmacologically or removing the Pre-N terminal 57 residues from full-length Grp94, OLF aggregation rates revert to those seen for OLF alone, but only pharmacological inhibition rescues co-aggregation. The Grp94-OLF interaction is below the detection limit of fluorescence polarization measurements, but chemical crosslinking paired with mass spectrometry analyses traps a reproducible interaction between OLF and the Grp94 N domain, as well as between OLF and the Grp94 M domain. The emerging molecular-level picture of quinary interactions between Grp94 and myocilin points to a role for the far N-terminal sequence of the Grp94 N domain and a cleft in the M domain. Our work further supports drug discovery efforts to inhibit these interactions as a strategy to treat myocilin-associated glaucoma.

## Introduction

**Figure 1:**
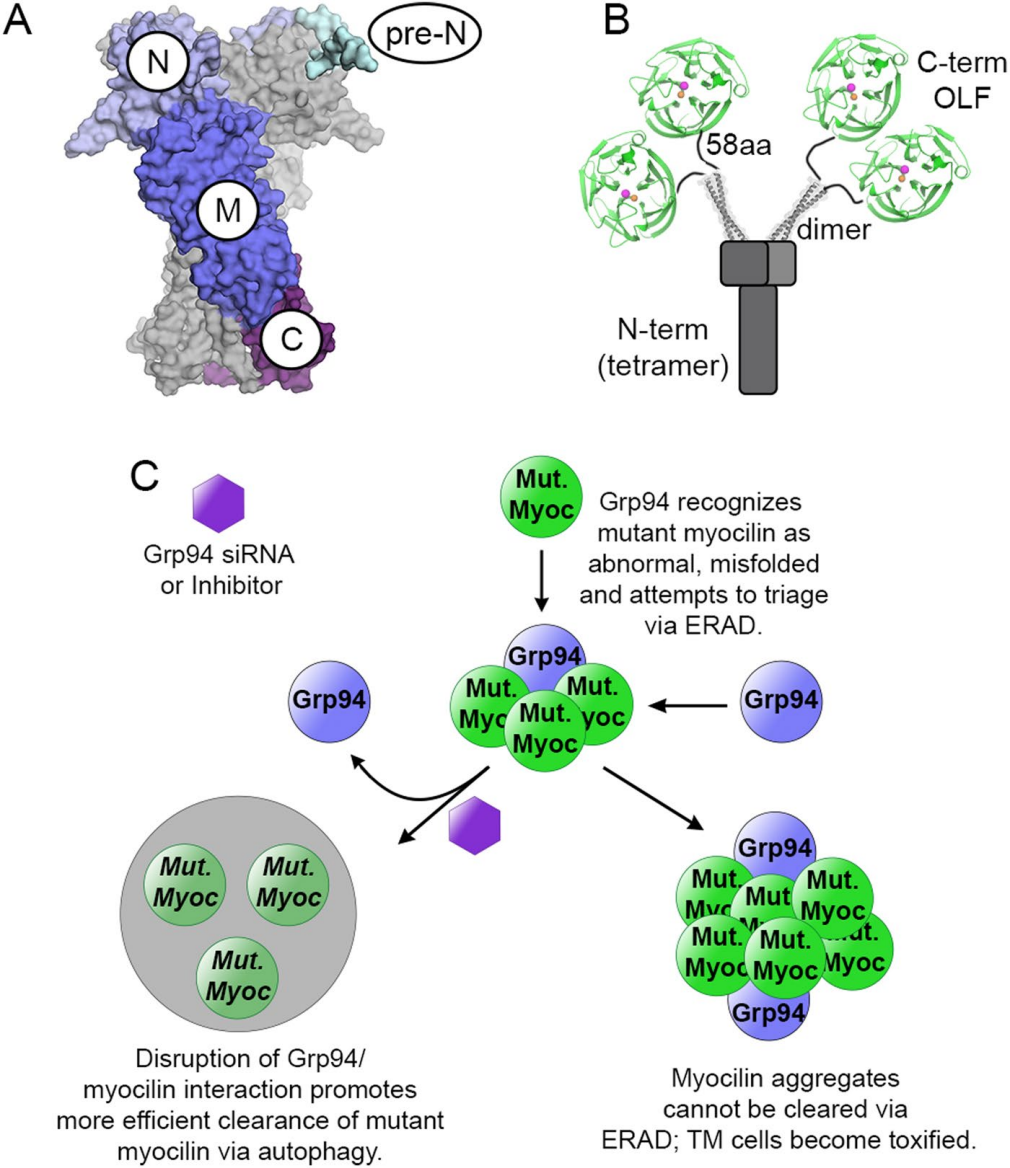
Structural features of and interaction model for Grp94 and myocilin. (**A**) Model of Grp94 highlighting its structural domains: Pre-N subdomain, cyan; N-terminal ATP-binding domain (N), light blue; middle domain (M), blue; C-terminal dimerization domain (C), purple. The second monomer of the obligate homodimer is shaded gray for clarity. (**B**) Schematic of myocilin structural organization emphasizing the C-terminal olfactomedin (OLF) domain in green. Colored spheres represent calcium (orange) and potassium (magenta) ions. Models are not drawn to scale. Models are from structures with PDB-ID codes 5ULS (Grp94) and 4WXQ, 5VR2 (myocilin). (**C**) Model of Grp94 involvement in myocilin aggregation and glaucoma-associated cellular toxicity. Grp94 is recruited by misfolded, aggregating mutant myocilin via the ERAD system, but counterproductively facilitates aggregation and preserves toxic aggregates; preventing the Grp94/myocilin interaction is a viable method of rescuing cells from cytotoxicity via alternative clearance mechanisms. Myoc = myocilin.

In the cell, the molecular chaperone network assists in protein folding and triages misfolded proteins to maintain homeostasis under normal and stressed conditions. Defects in protein folding are associated with numerous diseases as well as with the normal aging process^1^. The heat shock protein 90 (Hsp90) family of chaperones is a ubiquitous player in the late stages of client protein folding. Hsp90s identify destabilized or misfolded proteins to prevent their aggregation; they do not refold such proteins, which is an activity reserved for other chaperone systems^2^. Hsp90 paralogs, which are expressed in different compartments of the cell^3^, have a client base involved in signaling pathways that control cell homeostasis, growth, proliferation, differentiation, and cell death. For well-studied cytosolic Hsp90 family members, which have >500 known clients^4^, co-chaperone and client binding studies have revealed a case-specific, yet broad interaction surface that recognizes remaining exposed hydrophobic patches of largely-folded or intrinsically disordered clients^3^. By contrast, details of chaperone biology and mechanism of the endoplasmic reticulum (ER)-resident paralog glucose regulated protein 94 (Grp94, Fig. 1A), which has a short, selective client list consisting of proteins destined for secretion or cell surface localization, remain unclear^5^.

Grp94 recognizes mutant myocilin^6^, an extracellular protein expressed throughout the body^7^ but at high levels in the trabecular meshwork extracellular matrix tissue of the eye^8,9^. Mutations in the myocilin C-terminal olfactomedin domain (OLF, Fig. 1B) are causative for ocular hypertension and an accelerated timeline for the blindness disease glaucoma^10,11^. Unlike a typical client, stability-compromised mutant myocilins^12,13^ are not triaged for degradation by Grp94 (Fig. 1C), but rather co-aggregate with the chaperone, leading to further accumulation^14,15^, ER stress, and cytotoxicity^6,16–18^ that ultimately propagate to glaucoma-associated vision loss^19,20^. Mutant myocilin is degraded^6,21–24^ when cellular levels of Grp94 are reduced using siRNA, as well as when Grp94 is inhibited pharmacologically in cells and in the myocilin-glaucoma mouse model^18^. This phenotype is supported by *in vitro* assays using purified proteins, wherein Grp94 co-aggregates with OLF and also accelerates OLF fibrillization, but Grp94 inhibitors targeting its N-terminal ATP binding site revert OLF aggregation rates and promote Grp94 solubility^21,23^. Consequently, inhibition of Grp94 holds promise as a therapy to treat myocilin-associated glaucoma^21–23,25^ by preventing its toxic gain-of-function—accumulation in cells—as the absence of myocilin in humans^26^ or mice^27^ does not lead to glaucoma.

To better understand the molecular factors governing the interaction between Grp94 and OLF, we dissected Grp94 into component domains N, NM, MC (Fig. 1A). In our OLF aggregation assay, aggregation enhancement is localized to the Grp94 N domain (Grp94_N_), whereas other constructs (Grp94_NM_, Grp94_MC_) exhibit neutral or stabilizing effects on OLF aggregation. Biophysical and bioanalytical analysis indicate a transient interaction between OLF and Grp94 M and N domains. OLF aggregation rates revert to those seen for OLF alone when the isolated N domain is inhibited pharmacologically or when the terminal 57 residues are removed from full-length Grp94, but only pharmacological inhibition rescues co-aggregation. Collectively, our data inform a model of the aberrant Grp94/OLF interaction, wherein an initially transient interaction with a chaperone goes awry.

## Results

### Grp94 domains have contrasting effects on OLF aggregation

Grp94 is a dynamic chaperone whose overall architecture imparts the conformational plasticity required for client recognition. Full-length Grp94 (Grp94_FL_) is composed of three domains (Fig. 1A). Grp94_N_ houses a functionally-relevant ATP binding site, an unstructured linker, as well as a flexible region at the N terminus (Pre-N). The linker is highly charged and thought to play a role in calcium binding and regulation within the ER^28,29^ while providing flexibility critical for ATPase activity and regulating client/co-chaperone binding^30–33^. The Pre-N subdomain contributes to client maturation and regulates the rate of ATP hydrolysis^29^. The middle (M, Grp94_M_) domain contains residues required for ATP hydrolysis, and conformation-dependent interactions with Grp94_N_. The C-terminal (C, Grp94_C_) domain contains residues required for protein-protein interactions that facilitate homodimer assembly.

To identify the interactions responsible for recognizing misfolded OLF, well-behaved Grp94 domain constructs Grp94_N_ (residues 22–337), Grp94_NM_ (residues 22–594), and Grp94_MC_ (residues 336–765) were prepared as in Dollins *et al*.^34^ and tested for their ability to modulate the aggregation rate of OLF. Our aforementioned *in vitro* aggregation kinetics assay involves incubating wild-type OLF at 42 °C in PBS (see Experimental Procedures), which converts OLF to a thioflavin T (ThT)-positive aggregate over the course of 24–96 h^21,23,35^. Under these heat-shock mimicking conditions, OLF is in an early unfolding regime that mimics the partially folded state of glaucoma-associated OLF variants, and end-point aggregates have been vetted extensively as amyloid-like^35^; destabilized OLF variants and full-length myocilin (Fig. 1B) are not available in preparative yields required for the assay. The presence of Grp94_FL_ at stoichiometric (1:1) or sub-stoichiometric (1:10) ratios in the assay accelerates OLF aggregation, and the two proteins co-aggregate, a phenomenon that is reverted with Grp94-directed inhibitors^21,23^.

**Figure 2:**
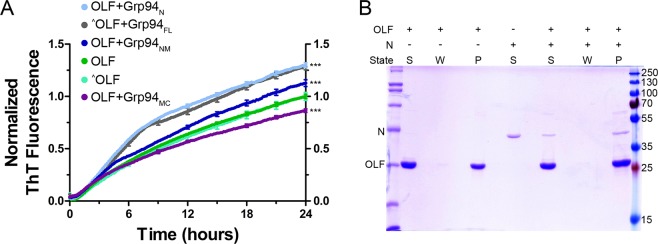
The N-terminal domain of Grp94 is responsible for the aberrant Grp94/OLF protein-protein interaction. (**A**) Grp94_N_ enhances the rate of OLF aggregation, whereas Grp94_MC_ appears to stabilize OLF against aggregation as indicated by ThT fluorescence. Results represent the average of 20 (OLF + Grp94_N_), 12 (OLF + Grp94_NM_), and 12 (OLF + Grp94_MC_) replicates from at least 2 biological replicates. The ^^^ symbol represents data presented previously^23^; ***(p < 0.0001) represents statistically significant differences relative to OLF at 24 hours. (**B**) The Grp94 N-terminal domain and OLF co-aggregate over the course of the aggregation assay in (**A**). S = supernatant, W = wash, and P = pellet/aggregate. See Fig. S1 for full four-day kinetics assay data, and Fig. S2 for additional co-aggregation SDS-PAGE gels for the remaining domain constructs.

We conducted our aggregation kinetics assay using Grp94_N_, Grp94_NM_, or Grp94_MC_ in sub-stoichiometric (3 μM Grp94: 30 μM OLF, Figs 2A and S1) or stoichiometric (30 µM Grp94: 30 µM OLF, Fig. S3) ratios. Results reveal that Grp94_N_ recapitulates the OLF aggregation rate enhancement seen previously with Grp94_FL_^21,23^. Aggregation rate enhancement is apparent early in the kinetics read (Figs 2A and S3A) and high levels of ThT fluorescence persist through the 4-day experiment (Figs S1 and S3B). SDS-PAGE analysis of the insoluble material at the conclusion of the experiment shows co-aggregation of Grp94_N_ and OLF (Fig. 2B), as observed with Grp94_FL_^21,23^. Consistent with rate enhancement, the presence of Grp94_N_ results in a ~30% increase in the amount of aggregated OLF compared to controls lacking Grp94 (Table 1).

**Table 1:** Impact of Grp94 domains on OLF aggregation.

Grp94 domain	% Soluble OLF	
	OLF-only	OLF + Grp94 domain
N	45.6 ± 13.9	32.5 ± 12.0
NM	55.0 ± 1.8	55.2 ± 1.1
MC	55.7 ± 1.9	64.6 ± 2.4

Data represent the average of at least two aggregation assay experiments.

Different effects on OLF aggregation are seen in the presence of Grp94_NM_ and Grp94_MC_ compared to Grp94_N_ or Grp94_FL_. Grp94_MC_, a homodimer in solution, modestly stabilizes OLF aggregation. This is evident in the slower initial rate and lower ThT fluorescence observed throughout the kinetics experiment (Figs 2A and S1) and corresponds to ~16% less OLF incorporation in the insoluble pellet (Fig. S2A, Table 1). Grp94_NM_ appears to have an intermediate effect on OLF aggregation rate (Figs 2A and S1). Grp94_NM_ co-aggregates with OLF, but does not change the relative ratio of soluble versus aggregated OLF (Fig. S2B, Table 1). This result is consistent with the interpretation that the N and M domains recognize and compete for OLF binding. In sum, based on aggregation experiments, the aggregation rate enhancement seen for OLF in the presence of Grp94 appears to be due to an interaction with Grp94_N_, but not Grp94_M_ or Grp94_C_ domains (Fig. 2A).

### Chemical crosslinking and mass spectrometry (MS) reveals interactions between OLF and Grp94 N and M domains

To further define interaction interfaces between Grp94 and OLF at the molecular level, we conducted amine-reactive chemical crosslinking coupled with MS analysis. To initially detect and optimize crosslinking, OLF was dansylated (OLF_d_) and crosslinked with Grp94_FL_. We used the chemical crosslinker BS^3^, which has a reasonable inter-residue C_α_-C_α_ capture space of 26–30 Å^36^. Crosslinking with stable isotope-labeled BS^3^_d0/d4_ to facilitate MS analysis resulted in discrete bands observable by SDS-PAGE using both Coomassie blue staining and dansyl fluorescence (Fig. S4, Table S1). In parallel, bands from a non-dansylated replicate were analyzed by in-gel digestion and LC-MS; crosslinked peptides could be distinguished by a 4 dalton mass shift signature in the MS spectrum (see Experimental Procedures). A total of 58 homomeric Grp94_FL_ crosslinks (unique peptide-peptide linkages) were detected across two independent experiments, which were consistent with published structures of Grp94, and mimicked previously reported homomeric crosslinks^37^ (Table S2-tab 1).

**Figure 3:**
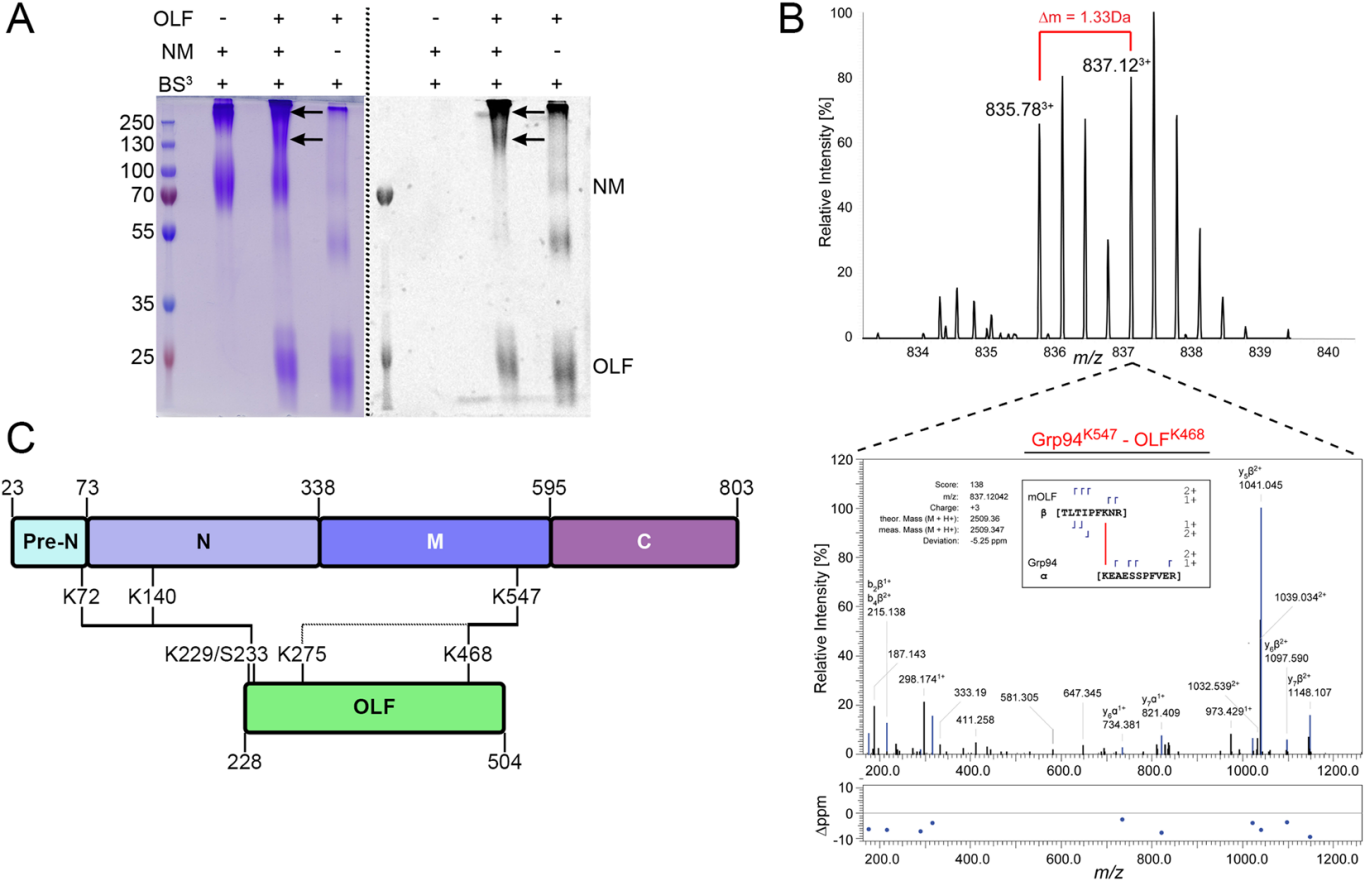
Mapping the Grp94/OLF protein-protein interaction interface. (**A**) Coomassie blue (left) and dansyl fluorescence (right) visualization of crosslinking reaction products (arrows) as seen by SDS-PAGE for the coupling of Grp94_NM_ with OLF. Dashed line distinguishes different visualizations of same gel. See Fig. S5 for complementary reactions with Grp94_N_ and Grp94_MC_. See Fig. S11B for uncropped gel images. (**B**) MS spectrum of crosslinked peptides containing the Grp94^K547^-OLF^K468^ linkage (top). HCD fragmentation spectrum and fragment mass match accuracy (1 of 9 PSMs observed) with y- and b-ion assignments (inset) consistent with the crosslinked peptides shown (bottom). Data correspond to the sample gel shown in Fig. S6B; refer to Table S2-tabs 2-3 for additional details. (**C**) Map of the interaction interfaces between Grp94 and OLF identified by mass spectrometry. Protein domains demarcated by first amino acid residue in the domain (top, Grp94; bottom, OLF). Colors match those of the models in Fig. 1.

We did not confidently observe any heteromeric crosslinks between Grp94_FL_ and OLF despite the requirement for such linkage to generate the molecular weight shifts we observed by SDS-PAGE. Suspecting that this could result from sample complexity due to the size of Grp94_FL_ compounded by the inherent difficulty of detecting low-frequency crosslink events, we next used BS^3^_d0/d4_ to evaluate crosslinking between OLF and Grp94_N_ (including Pre-N), Grp94_NM_, or Grp94_MC_ domains independently (Figs 3A, S5, S6, Table S1). A total of 78 protein-protein crosslinks (unique peptide-peptide linkages) were observed across all experiments, four of which corresponded to unique heteromeric crosslinks between Grp94 domains and OLF. Heteromeric crosslinks revealed three interaction sites on both Grp94 (K72, K140, K547) and OLF (K229/S233, K275, K468) (Figs 3B,C and S7, Table S2-tabs 2-3), derived from experiments with Grp94_N_ and Grp94_NM_.

**Figure 4:**
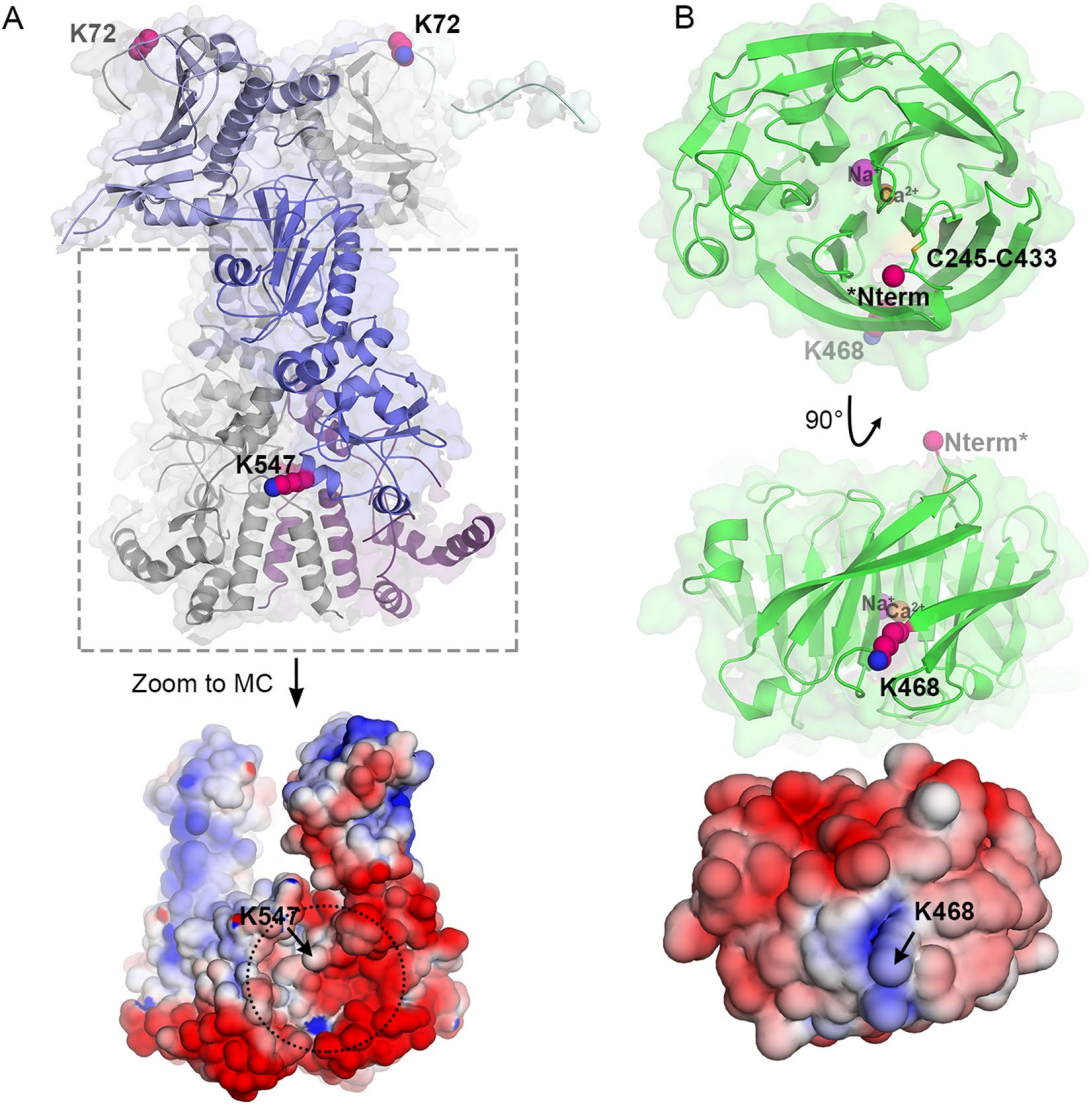
Model of Grp94 protein-protein interaction sites with OLF. (**A**) Top: structural model of Grp94 highlighting both the aberrant (K72) and stabilizing (K547) interaction sites detected by crosslinking/MS (see Table S2-tabs 2-3). Bottom: electrostatic surface potential view of boxed MC region; Grp94_MC_ model PDB-ID: 2O1T, which contains 18 additional, largely acidic C-terminal residues compared to 5ULS shown above (also see Fig. S8). The proposed OLF recognition surface is identified by dotted circle. (**B**) Top: top-down perspective of OLF β-propeller, with unstructured N-terminal aggregation-associated Grp94 recognition site marked as Nterm*. Middle: side-on view of OLF with stabilizing interaction residue (K468) featured. Bottom: electrostatic surface potential of OLF in same pose as middle panel, indicating a positively-charged surface. The surface potential is colored negative (red, −5 kT/e^−^) to positive (blue, 5 kT/e^−^). The remaining color scheme and models shown are the same as in (Fig. 1) unless otherwise specified.

The most frequently observed heteromeric crosslink corresponds to a linkage between Grp94^K547^ in the M domain and surface-exposed OLF^K468^ in the β-propeller, for which 9 total peptide spectral matches (PSMs) were acquired in experiments using Grp94_NM_ (Fig. 3B,C, Table S2-tabs 2-3). Grp94^K547^ is nestled in a bowl-like cleft (~30 Å diameter, ~17 Å deep) between the M and C domains of one Grp94 protomer and the C domain of a second protomer, surrounded by a largely negatively-charged surface (Fig. 4A, bottom). This site is distinct from the MC luminal surface previously postulated to bind unfolded polypeptides^29^. Comparison of the available structures of Grp94 and its domains suggests that this cleft should be present regardless of the overall Grp94 conformation (Fig. 4A bottom, Fig. S8). Moreover, this cleft can accommodate OLF^K468^, which together with OLF^R470^, form the only positively-charged patch on the OLF surface (Fig. 4B middle, bottom and Fig. S9).

The next most frequent crosslink was observed between Grp94^K72^ within Grp94_N_ and OLF^S233^, the N-terminal residue of the OLF construct (2 PSMs; Fig. S7A; Table S2-tabs 2-3). This region constitutes a linker prior to the start of the structured OLF domain at Gly244 (Fig. 4B). Although it is possible that crosslinking to the unstructured N-terminus of our OLF construct is a fortuitous event owing to flexible regions on each protein, there is reason to propose this result is meaningful. First, Grp94^K72^ is found one residue before the start of the first strand of structured N domain (Fig. 4A) and was the only observed chemical crosslink in Pre-N. Compared to the two other primary amines in the unstructured Pre-N available for crosslinking, Grp94^K72^ is more spatially restricted and thus the least probable to form nonspecific interactions. Second, the result is consistent with the observation that aggregation enhancement of OLF in the presence of Grp94 is abolished when pharmacological inhibitors targeting the ATP binding site are present^21,23^. Namely, this scenario is analogous to AMP-PNP binding in which Pre-N residues organize and interact with the structured N domain of an opposing protomer^29^, making these residues inaccessible to OLF.

Both of the observed crosslinks suggest that Grp94 is inspecting OLF near its N and C termini, which form the canonical molecular clasp that confers the native-state closed-circular arrangement for β-propellers such as OLF. In OLF, there is a disulfide bond nearby (Cys245-Cys433. Fig. 4B, top) that is needed to further stabilize^13^ the toroidal structure^38^. The molecular clasp is close to the proposed amyloidogenic core Val426-Tyr442^35^, near a mobile loop^38^. In the case of the apparently protective M domain, inspecting this region of the protein would confirm the presence of native state attributes; in the case of the N domain, proximity to the amyloid core might facilitate co-aggregation.

Lastly, two additional low-frequency crosslinks (1 PSM) support the two main crosslinks described above. These crosslinks were observed between Grp94^K140^ and OLF^K229^ and Grp94^K547^ and OLF^K275^ (Fig. S7B,C, Table S2-tabs 2-3). Grp94^K140^ is close (~32 Å C_α_-C_α_ in the apo Grp94_N_ structure) to Grp94^K72^, providing further evidence of an interaction between this region on Grp94 and the unstructured N-terminal region on OLF. The Grp94^K547^-OLF^K275^ crosslink, the highest scoring of the heteromeric crosslinks, is consistent with the more frequently detected Grp94^K547^-OLF^K468^ crosslink modeled in Fig. S9. OLF^K275^ lies on a long loop (14 amino acids) on the same face of the β-propeller as the N-terminus and disulfide bond, and, as modeled, is ~32 Å from Grp94^K547^.

### Analysis of crosslinking and MS further reveals OLF dimerization

A common feature across controls in all crosslinking experiments was the formation of homo-oligomers of OLF (Figs 3A, S4A, S5 and S6). The OLF domain is a monomer in solution even at high concentrations^12^, but in the context of the full-length dimer-of-dimers tetramer of myocilin (Fig. 1B), two pairs of OLFs are connected by linkers to the coiled-coil domains, which confer the dimer and tetramer^39^. The predominant species captured by crosslinking was an apparent dimer at ~50–55 kDa, close to the expected mass of ~62.5 kDa (Table S1). Analysis of crosslinked peptides (Table S2-tabs 4-5) reveals two binding surfaces, but these are remote from one another. Unfortunately, crosslinking/MS cannot parse intra- from inter-chain crosslinks for homomeric interactions and these data do not resolve a singular OLF-OLF protein-protein interaction interface. Nevertheless, the finding that OLF forms a transient dimer is consistent with our structural model for full-length myocilin and lends support for the notion that OLF interactions with itself and likely other extracellular matrix components drive the still-elusive^11^ myocilin function.

### Removal of the Grp94 Pre-N sequence or inhibition of Grp94_N_ pharmacologically reverts OLF aggregation rate enhancement

**Figure 5:**
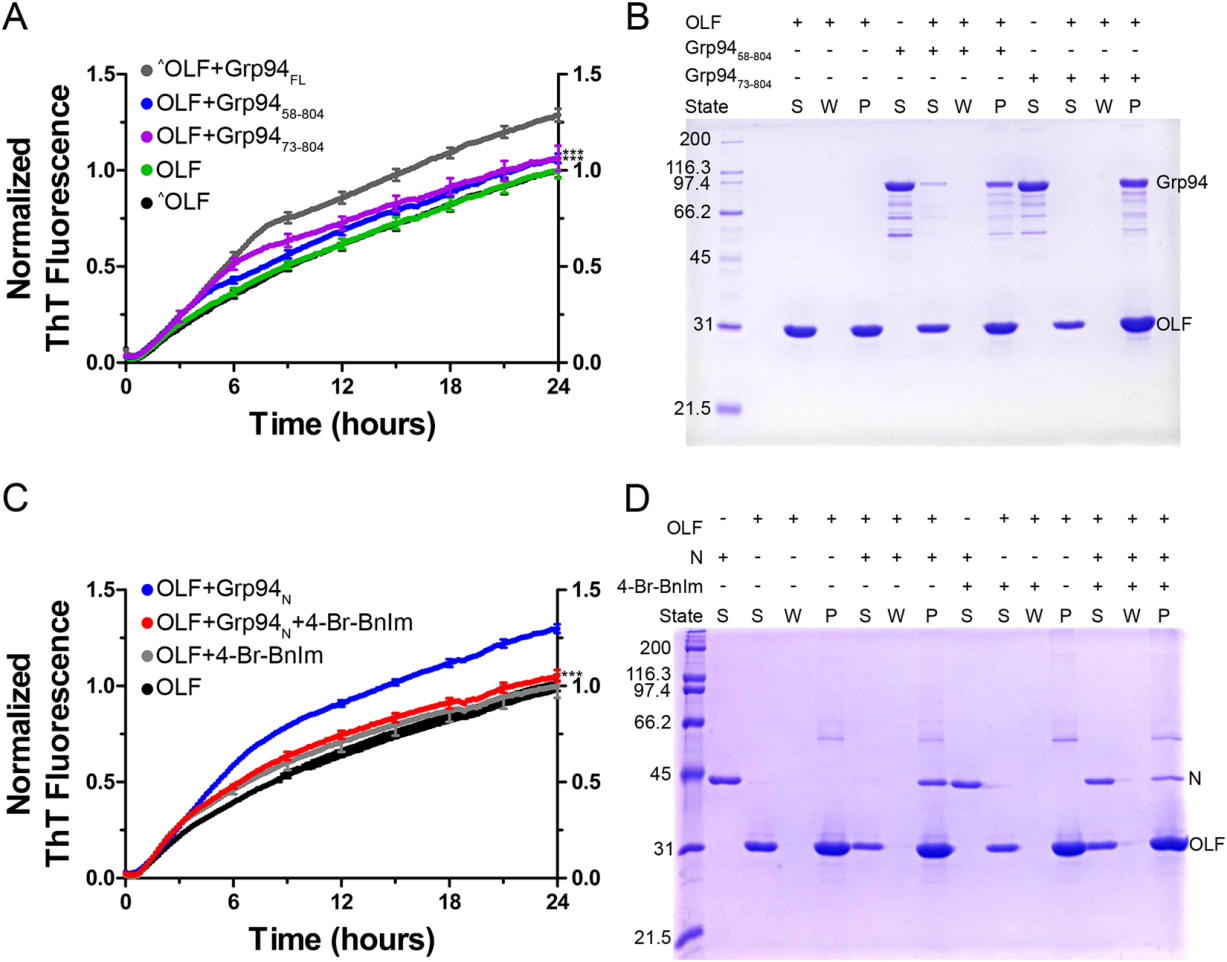
The Pre-N region and conformational state of Grp94_N_ are critical for the aberrant interaction with myocilin OLF. (**A**) Truncation of the N domain of Grp94 eliminates its capacity to enhance the rate of OLF aggregation; residues 22-57 appear crucial to the interaction. Results represent the average of 12 replicates (for both OLF + Grp94_58-804_ and OLF + Grp94_73-804_) from 2 biological replicates. The ^^^ symbol represents data previously published^23^; ***(p < 0.0001) represents statistically significant differences relative to OLF + Grp94_FL_. (**B**) Despite diminished effects on OLF aggregation, the majority of the Grp94 truncation variant proteins still co-aggregate with OLF. S = supernatant, W = wash, P = pellet/aggregate. (**C**) Treatment of Grp94_N_ with Grp94-specific inhibitor 4-Br-BnIm mitigates its interaction with OLF. The traces for Grp94_N_ (for comparison) are the same as in Fig. 2A; results for OLF + Grp94_N_ + 4-Br-BnIm represent the average of 9 replicates from 2 biological replicates. ***(p < 0.0001) represents statistically significant differences relative to OLF + Grp94_N_. D) Grp94_N_ is partially rescued from its co-aggregation fate with OLF in the presence of inhibitor 4-Br-BnIm. Abbreviations given in (**B**).

Together, our kinetics aggregation assays and chemical crosslinking/mass spectrometry analyses suggest the Pre-N region of the Grp94 N domain is responsible for the protein-protein interaction with OLF leading to accelerated aggregation. To bolster this result, first we conducted the aggregation assay with Pre-N truncation variants of Grp94, Grp94_58-804_ and Grp94_73-804_ as characterized by Huck *et al*.^29^. Neither variant enhanced the rate of OLF aggregation (Fig. 5A), localizing the deleterious interaction with OLF to unstructured Pre-N residues 22–57. Interestingly, Grp94_58-804_ and Grp94_73-804_ co-aggregated with OLF (Fig. 5B), suggesting that rate enhancement and co-aggregation are not coupled.

Second, to test whether inhibition of Grp94_N_ at its nucleotide binding site rescues coaggregation as with Grp94_FL_^21,23^, we added 25 µM 4-Br-BnIm^21–23^ to the aggregation assay with OLF + Grp94_N_. Indeed, inhibition of the N domain alone reverts OLF aggregation to baseline levels (Fig. 5C). Moreover, unlike truncated Grp94 variants (Fig. 5B), co-aggregation of Grp94_N_ and OLF is substantially reduced in the presence of 4-Br-BnIm (Fig. 5D). In the absence of inhibitor, ~13% of Grp94_N_ remains soluble at the conclusion of the assay, compared to ~65% in the presence of the inhibitor.

### Initial interactions between Grp94 and folded OLF are transient or involve low population conformational states of Grp94 and/or OLF

To further bracket the strength of interaction between Grp94 and OLF, fluorescence polarization experiments were performed with OLF_d_ containing the glaucoma-associated D380A variant (OLF_d_^D380A^), a less stable (T_m_ = 46.6 °C versus for 52.2 °C wild-type OLF) protein with no central Ca^2^^+^ ^13,40^. Titrating Grp94 into OLF_d_^D380A^ at a ratio of up to ~10,000 Grp94 dimer:1 OLF (93 μM Grp94 dimer: 0.01 μM OLF) at room temperature yielded no response after background subtraction (Fig. S10). This experiment adds to the growing body of evidence, including low frequency crosslinking (Fig. S4) and unproductive pull down experiments^21^ suggesting that the initial interaction between Grp94 and OLF is a low proabability event. Only when OLF protein is primed for assembly into amyloidogenic material is the interaction with Grp94 irreversible as the two proteins co-aggregate.

## Discussion

In this study, we characterized the molecular interactions between Grp94 and OLF leading to their coaggregation, revealing a prominent role for the Pre-N region of the Grp94 N domain. Unlike cytosolic Hsp90s with many clients and co-chaperones, the current client list for Grp94 is small and selective. The list includes primarily β-sheet-containing proteins destined for cell surface expression or secretion^41–45^. Proposed new additions to the Grp94 client list are two olfactomedin domain-containing proteins, myocilin and latrophilin-1, the latter of which is an olfactomedin-containing GPCR^46^. Little is known about how Grp94 interacts with its clients, and where ATP hydrolysis fits into the client recognition and chaperoning cycle. Some bona fide clients^29,47^ bind Grp94_C_ residues 652–678 within the interior surface of the dimer, but immunogenic peptides are reported to bind Grp94_N_, in an activity seemingly unrelated to chaperoning^48^. Thus, client recognition by Grp94 remains an open question.

We found that Grp94 N and M domains both interact with OLF, with opposing effects on OLF aggregation. Whereas the interaction of OLF with Grp94_N_ promotes aggregation, the interaction with M appears to be protective, and C appears inert. The interaction we detected most was in the apparent protective mode, as might be expected for a chaperone recognizing a client. Contact between OLF and Grp94_N_ was also observed, albeit with less frequency. One possibility is that this interaction occurs briefly and stochastically with the fully folded OLF. Another possibility more consistent with the crowded^49^, destabilizing^50^ environment of the ER, is that the interaction is not transient but instead is conformationally selective for a low-population, partially-folded OLF state. We showed previously that such populations of OLF are primed for facile amyloid formation^35^, converting an initially weak interaction to a long-lived association, namely, a subcellular phase transition^51^.

Even though the OLF/N interaction appears to involve the largely unstructured Pre-N region adjacent to the structural N domain, only inhibition at the nucleotide binding site of the N domain, isolated (this work) or in full-length Grp94^21,23^, can both revert aggregation rates and rescue Grp94 from coaggregation with OLF. As observed in the structure of Grp94 in the closed conformation with AMP-PNP bound in the nucleotide binding pocket (Figs 1A and 4A)^29^, Pre-N is sequestered in the dimer interface. Pre-N and other surfaces exposed in the predominantly-populated open conformation^34^ are also likely not accessible to OLF when Grp94 adopts a closed conformation. Without the dimerization C domain, N domain is a monomer in solution, but an inhibitor-bound NM construct of the mitochondrial Hsp90 paralog Trap1 crystallized as a closed dimer^52^, supporting the possibility that the isolated N domain can sequester key problematic surfaces for OLF. Finally, in support of the hypothesis that Grp94 conformational states are relevant to the aberrant interaction with OLF, we also tested two glaucoma-causing variants OLF^D380A^ or OLF^I499F^ for their rate enhancement with Grp94_N_ (data not shown), experiments that had to be conducted at 32 °C and 28 °C, respectively to match the aggregation kinetics of wild-type OLF at 42 °C. At these low temperatures we did not observe a rate enhancement, likely because we limited the conformational plasticity of the chaperone and its domains.

Grp94 N and M domains could interact with OLF simultaneously, in a particular sequence, or the interactions could be mutually exclusive. The largely unstructured Pre-N region and M could interact with a single OLF domain within myocilin at the same time because of the flexibility introduced by the linker connecting the N and M domains. Alternatively, since interactions with M domain appear to attempt to stabilize OLF against aggregation, M may serve as the initial docking site, perhaps priming the subsequent aberrant interaction with Pre-N, in a failed attempt at ERAD. Related, conformational changes may occur upon OLF binding, as seen in studies of *E*. *coli* Hsp90^53^. This could alter the extent to which Grp94 is open or closed and thus the relative distance of OLF to each of the identified Grp94 interacting sites. The apparent recognition cleft we identified on M is exposed in structures of Grp94_FL_ in partially closed (PDB code 2O1V) and fully closed (PDB code 5ULS) conformations, implying that a range of chaperone states could bind OLF. Finally, given the transient nature of the initial interaction juxtaposed with the irreversible nature of amyloid aggregation^54,55^, it is possible that all statistically-driven quinary interactions involving the Grp94 N domain lead to irreversible co-aggregation, regardless of proper attempts for chaperone function at the M domain.

In sum, interactions between OLF and Grp94 are multifactorial and initially transient or weak. The Pre-N subdomain appears culpable for the aberrant interaction seen previously between Grp94_FL_ and mutant/misfolded myocilin that forms glaucoma-relevant cytotoxic co-aggregates. There is also a recognition surface within the M domain that appears to stabilize OLF from aggregation. Further temporal and molecular resolution of this unusual interaction remain to be elucidated. By pursuing a molecular understanding of how Grp94 interacts with myocilin, we had hoped to identify a unique protein-protein recognition surface that could provide an alternative target to the N domain ATP binding site common to all Hsp90s for disrupting the Grp94/myocilin interaction^25^, and would circumvent the challenges posed in achieving selectivity for Grp94^56^. Our study supports continued efforts to block the ATP binding site but broadens the possibility to any molecule that drives Grp94 to a closed state in which Pre-N is occluded.

## Methods

### Recombinant protein expression and purification

Grp94_FL_ (*Homo sapiens*, residues 22–803 with an N-terminal hexahistidine (6xHis) tag followed by thrombin and tobacco etch virus (TEV) protease cleavage sites, cloned in a pET28a vector) was expressed and purified as previously described^23^. Grp94_N_ and Grp94_NM_ plasmids were prepared employing site-directed mutagenesis (QuikChange Lightning Site-Directed Mutagenesis Kit (Agilent Technologies)) on the Grp94_FL_ template, installing STOP codons after positions 337 and 594, respectively, using primers designed by Eurofins MWG Operon; see Table S3 for mutagenesis primers. Plasmid fidelity was assessed with DNA sequencing by Eurofins MWG Operon. Typically, Grp94_N_ and Grp94_NM_ proteins were expressed in *E*. *coli* BL21(DE3) cells cultured in Luria-Bertani broth (BD) supplemented with 50 mg L^−1^ kanamycin sulfate (Sigma-Aldrich). The cells were grown at 37 °C and 225 rpm until OD_600_ = 0.6–0.8 was reached, at which point they were induced with 1 mM isopropyl-β-D-1-thiogalactopyranoside (IPTG, GoldBio). Cells were allowed to shake for an additional 4 hours prior to harvesting via centrifugation (10 minutes at 4,420 × g, 4 °C) and flash cooled in liquid nitrogen. To improve expression yield, cells were sometimes also cultured in Superior Broth (US Biological); see Supplemental Information.

Immediately before purification of Grp94_N_ and Grp94_NM_, cells were thawed at room temperature and resuspended in Tris wash buffer (50 mM Tris, 0.5 M NaCl at pH 8.0) supplemented with deoxyribonuclease I (DNase I, Sigma-Aldrich) and protease inhibitor cocktail (cOmplete Tablets EDTA-free, Roche). Cells were lysed by double passage through a French Press. The lysate was clarified by ultracentrifugation (1 hour at 164,700 × g and 4 °C). Grp94_N_ and Grp94_NM_ were isolated with a HisTrap HP 5 mL column (GE Healthcare) equilibrated with Tris wash buffer supplemented with 40 mM imidazole, and the proteins were eluted with a gradient of 40–500 mM imidazole. Eluted Grp94_N_ and Grp94_NM_ were subjected to size-exclusion chromatography purification (HiLoad 16/60 Superdex 75 preparatory grade column) equilibrated with phosphate buffer (PBS, 10 mM sodium phosphate dibasic, 10 mM potassium phosphate monobasic, 0.2 M NaCl at pH 7.2). Next, the N-terminal affinity tags were cleaved with overnight treatment (~16 hours) of 1:10 (w/w) TEV protease, and the histagged TEV protease was subsequently eliminated from the protein solution via refractionation on the 5 mL HisTrap HP column. Grp94_N_ and Grp94_NM_ were subjected to a final polishing step by size-exclusion chromatography on the HiLoad 16/60 Superdex 75 column.

Grp94_MC_ protein domain construct gene (residues 336–765, *Canis lupus familiaris* origin, 98.4% identity to human sequence for Grp94_MC_, 97.9% identity overall based on Clustal Omega alignment^57^) was synthesized and codon-optimized for *E*. *coli* expression by ATUM and cloned into a pMAL-c5X vector (New England Biolabs) so that it contained a TEV-cleavable N-terminal maltose binding protein (MBP), and a Factor Xa-cleavable C-terminus 6xHis tag. Grp94_MC_ was expressed in *E*. *coli* as described above. After cell harvest, the resulting cell paste was resuspended in PBS containing DNase I and protease inhibitor cocktail, and subjected to two rounds of lysis by French Press and ultracentrifugation as above. The MBP-Grp94_MC_ fusion protein was isolated using a 20-mL HR 16/10 column (GE Healthcare) packed with amylose affinity resin (New England Biolabs) and equilibrated with PBS with 1 mM EDTA. MBP-Grp94_MC_ was eluted by PBS supplemented with 1 mM EDTA and 10 mM maltose. MBP-Grp94_MC_ was further polished using a HiPrep 16/60 Sephacryl S-300 size-exclusion chromatography column (GE Healthcare) equilibrated with PBS. MBP was removed with TEV protease, followed by removal of TEV protease by the aforementioned 5 mL HisTrap column; Grp94_MC_ and TEV protease proteins were eluted with Tris wash buffer containing 0.5 M imidazole. Finally, Grp94_MC_ was separated from TEV protease by fractionation on a HiPrep 16/60 Sephacryl S-300 h column equilibrated with PBS.

Plasmids coding for Grp94 N-terminal truncation variants Grp94_58-804_ and Grp94_73-804_ (residues 58–804 and 73–804, respectively, *Canis lupus familiaris* origin, in pET22b with non-cleavable C-terminal 6xHis tag) were a gift from Prof. Daniel Gewirth (Hauptman-Woodward Medical Research Institute, University at Buffalo). Both protein constructs were expressed and purified in a manner analogous to Grp94_FL_^23^.

OLF (*Homo sapiens*, residues 228–504) was expressed and purified as previously described^35^. OLF^D380A^ was prepared with the MBP-OLF template by implementing site-directed mutagenesis as discussed above using previously published primers^12^. Mutations were confirmed by sequencing by Eurofins MWG Operon. OLF^D380A^ was purified in a manner analogous to wild-type OLF. The TEV protease was prepared in-house using the pRK793 plasmid^58^. For all proteins described in this manuscript, purity was assessed by SDS-PAGE analysis with Coomassie staining. Protein concentration was assessed using the absorption at 280 nm and a calculated extinction coefficient from ExPasy^59^.

### ThT fluorescence kinetics aggregation assay

A working stock of 200 μM ThT (Sigma-Aldrich) was prepared in PBS, diluted from a 1 mg mL^−1^ master solution in nanopure water. Concentrated protein stocks were prepared in PBS, subjected to centrifugation (5 minutes at 4 °C and 17,000 × g) and quantified immediately prior to assay setup. All assay samples contained 10 μM ThT and were prepared in PBS as master mixes, with the Grp94 protein/domains at 3 μM (or 30 µM, as in Fig. S3) and OLF at 30 μM, when present. When Grp94 inhibitor 4-Br-BnIm (gift from Prof. Brian Blagg, University of Notre Dame) was included, it was added from a 20 mM stock in DMSO to a final concentration of 25 µM and overall DMSO concentration of 0.5% (v/v). Master mixes were dispensed in 150 μL volumes into 96-well microplates (black well, black bottom, medium binding, Grenier) at room temperature. Microplates were sealed with clear MicroAmp PCR film (Applied Biosystems) and loaded into a Biotek Synergy 2 plate reader at 42 °C. ThT fluorescence readings (λ_ex_ = 440 nm, λ_em_ = 485 nm) were sampled every 10 minutes for up to 96 hours. ThT fluorescence kinetics aggregation assay data presented in text represent averages of at least 2 biological replicates, with the specific number of analytical replicates described in the respective figure legends. Two-tailed, unpaired t-tests were conducted in GraphPad Prism software to evaluate statistical significance.

### Assessment of co-aggregation by SDS-PAGE

Following ThT aggregation assays, the 96-well microplates were removed from the plate reader and well content harvested. Identical samples were pooled in microcentrifuge tubes, subjected to centrifugation (10 min at 4 °C and 17,000 × g), and processed as described previously^21,23^. The resulting supernatant, wash, and pellet specimens were analyzed by 12% reducing SDS-PAGE; only samples containing OLF resulted in the formation of isolable aggregates/pellets. Gels were stained with Coomassie blue, destained, and bands quantified by densitometric analysis with ImageJ software (http://imagej.nih.gov/ij/).

### Modification of OLF with dansyl chloride

Chemical modification of OLF (wild-type or D380A) with dansyl chloride was adapted from ref.^60^ and the procedure was performed in darkness. A working stock of 100 mM dansyl chloride (Acros Organics) was prepared in dimethylformamide (DMF). A 100 μM stock of OLF in PBS was exchanged into 10 mM MOPS buffer at pH 8.4 using Amicon Ultra 0.5 mL centrifugal filter devices (Millipore). OLF was then transferred to a small glass vial equipped with a stir bar, diluted to a 1.5 mL volume and a concentration at or exceeding 100 μM OLF, and reacted with 2–5x molar excess dansyl chloride (total concentration DMF < 1%) for 1.5 hours at room temperature, with slow stirring. The reaction was quenched with addition of 10 mM Tris at pH 8.0. Unreacted fluorophore was removed by an Econo-Pac 10DG desalting column (Bio-Rad) equilibrated with PBS. Dansyl modification was confirmed by absorbance at 340 nm, MS (data not shown, ~2 dansyl modifications were obtained for OLF_d_ samples measured), and with fluorescent bands detected on SDS-PAGE gels (Figs 3A, S4A and S5).

### Chemical crosslinking

Grp94_FL_, Grp94_N_, Grp94_NM_, and Grp94_MC_ were crosslinked to OLF with the BS^3^ crosslinker. Working stocks of 50–70 mM BS^3^(d0) (Thermo Scientific) and BS^3^(d4) (ProteoChem) were dissolved in PBS immediately prior to initiating crosslinking reactions and were mixed 1:1 to yield BS^3^(d0/d4) to facilitate downstream mass spectrometric analysis of crosslinked products. Concentrated stocks (>100 μM) of Grp94 proteins and OLF (dansyl-modified and label-free) were prepared in PBS. Protein components were mixed in microcentrifuge tubes such that Grp94:OLF was 1:20 and Grp94_N_, Grp94_NM_, or Grp94_MC_:OLF was 1:1, with OLF at 100 μM. Controls consisted of Grp94_FL_, Grp94_N_, Grp94_NM_, and Grp94_MC_ -only or OLF-only samples. For Grp94_FL_, the chaperone was pre-incubated with OLF for 30 minutes at 42 °C prior to addition of crosslinker. BS^3^(d0/d4) was added last to the protein samples. Grp94_FL_-containing samples were treated with 50 μM BS^3^(d0/d4) for 1 hour at 42 °C. Grp94 domain-containing samples were dosed with 10 mM BS^3^(d0/d4) and incubated for 3 hours at 37 °C. Crosslinking reactions were quenched by addition of excess Tris at pH 8.0. Crosslinked products were mixed with reducing Laemmli buffer, boiled for 3 minutes, and run on 8- or 12% SDS-PAGE gels. BS^3^-treated sample gels containing OLF_d_ were first visualized with an Amersham Imager 600 to detect fluorescent gel bands prior to being stained with Coomassie blue. All gels were imaged with the Amersham Imager 600 after Coomassie blue staining. Dansyl-modified protein samples, reactions, and gel analyses were conducted in darkness to prevent photobleaching.

### Mass Spectrometry

Bands of interest were excised from the Coomassie-stained gels, cut into 1 mm squares and placed into a 1.5-mL Axygen tube. The gel pieces were destained through several incubations with a solution of 1:1 acetonitrile (ACN):50 mM ammonium bicarbonate (ABC) until clear/colorless, and then dehydrated with ACN. The gel samples were subsequently reduced by treatment with 10 mM dithiothreitol (DTT) for 30 minutes. Excess DTT was removed, and then the samples were alkylated with 20 mM iodoacetamide for 45 minutes in the dark. The gel slices were rinsed with 50 mM ABC and again dehydrated with ACN. Gel protein samples were then digested with MS-grade trypsin (Promega, Madison, WI) for 30 minutes on ice, followed by removal of unabsorbed trypsin solution. The gel pieces were then covered with 100 μL of 50 mM ABC and incubated at 37 °C overnight on a thermomixer (Eppendorf). After digestion, 70 μL ACN was added to the digest mixture and agitated for 10 minutes. The ABC/ACN was removed and saved in a fresh 1.5-mL vial followed by dehydration of the gel pieces with additional aliquots of ACN and collection of the solution into the fresh vial. The collected liquid was frozen at −80 °C overnight, and subsequently dried at 4 °C with a Centrivap (Labconco). The resultant digested peptides were then reconstituted using a solution of 5% ACN containing 0.1% formic acid (FA).

Peptide digest samples were analyzed with an Ultimate 3000 LC and a Q-Exactive Plus mass spectrometer from Thermo Fisher Scientific (Waltham, MA) run in data dependent mode (top 12) with HCD fragmentation. Samples were separated over an Acclaim Pep Map C18 RP column where mobile phase A (2% ACN with 0.1% FA) and mobile phase B (80% ACN with 0.1% FA) were used in a multistep gradient from 4% B up to 90% B over 150 minutes at a flow rate of 0.3 µL/min. MS spectra were acquired from 400–1800 m/z at 70 K resolution. Data were analyzed using Proteome Discoverer 2.0 and StavroX version 3.6.0.1^61^. All software-identified crosslinks were further manually interrogated to ensure the presence of clear BS^3^(d0/d4) doublets in the MS spectra, and assignments failing to pass this criterion were excluded from the final report. For crosslinked peptides in which specific site assignment was ambiguous, lysine residues resulting from missed trypsin cleavage are reported if there was no difference in the crosslink assignment probability between multiple positions.

### Protein structure modeling

Protein structure images were prepared with PyMOL (http://www.pymol.org). Surface electrostatics calculations were carried out with the PDB2PQR server^62^ with pH set to 7.2 (as in ThT kinetics aggregation assays) and visualized in PyMOL. Docking of OLF (PDB code 4WXQ) with Grp94_MC_ (PDB code 2O1T) was performed with the pyDockWEB server^63^, with a single interaction restraint imposed, that being the interaction of K547 of Grp94 with K468 of OLF.

### Fluorescence polarization

Samples for fluorescence polarization titration were prepared individually in microcentrifuge tubes and allowed to equilibrate for 3 minutes at room temperature (~25 °C) prior to fluorescence measurements. Each sample had a total volume of 60 μL and was composed of 0.01 μM OLF_d_^D380A^ with 0–185.6 μM Grp94 (concentration of monomer) in PBS. Grp94_FL_-only control samples were prepared in parallel. Samples were loaded into a Starna Cells 3 mm fluorescence cuvette, and fluorescence polarization measurements were acquired with an ISS PC1 photon counting spectrofluorometer controlled by the Vinci2 version 2.1 software package. The sample temperature was maintained at 25 °C throughout data collection with a Fisher Scientific isotemp refrigerated circulator (model 90) and a Quantum Northwest temperature control unit (TC 425). In addition, the source power was controlled with an ILC Technology illuminator power supply (model PS300-1). During data collection, samples were excited with 340 nm light and emission was monitored at 492 nm; both excitation and emission slits were set to 2.0 nm. Data were background-corrected and plotted with GraphPad Prism 5, with each data point representing the average of 15–30 scans.

## Supplementary information

Supplementary Information

Supplementary Table S2

## Data Availability

The datasets generated during and/or analyzed during the current study are available from the corresponding author on reasonable request.
