# Supplementary material for: Different Grp94 components interact transiently with the myocilin olfactomedin domain in vitro to enhance or retard its amyloid aggregation
Source: Sci Rep. 2019 Sep 4;9:12769. doi: 10.1038/s41598-019-48751-8 (PMC6726633; doi:10.1038/s41598-019-48751-8)
Supplement: Supplementary file 1 — Supplementary Information [file 41598_2019_48751_MOESM1_ESM.pdf]

**Supplementary Information**

# Different Grp94 components interact transiently with the myocilin olfactomedin domain *in vitro* to enhance and retard its amyloid aggregation

Dustin J. E. Huard, Alex P. Jonke, Matthew P. Torres, Raquel L. Lieberman

SUPPLEMENTARY METHODS

**Recombinant protein expression and purification.**
To bolster the yield of Grp94 protein/derived construct(s), BL21(DE3) *E. coli* cells were sometimes cultured in Superior Broth (US Biological) to which 50 mg $L^{-1}$ kanamycin sulfate was added. The cells were grown at 37 °C with shaking at 225 rpm, and once they reached $OD_{600}$ = 1.5, the temperature was reduced to 18 °C. The Grp94 construct-expressing cells were allowed to shake at 18 °C for up to 2 hours prior to addition of 1 mM IPTG, and then for an additional 16 hours (overnight) prior to harvest via centrifugation.

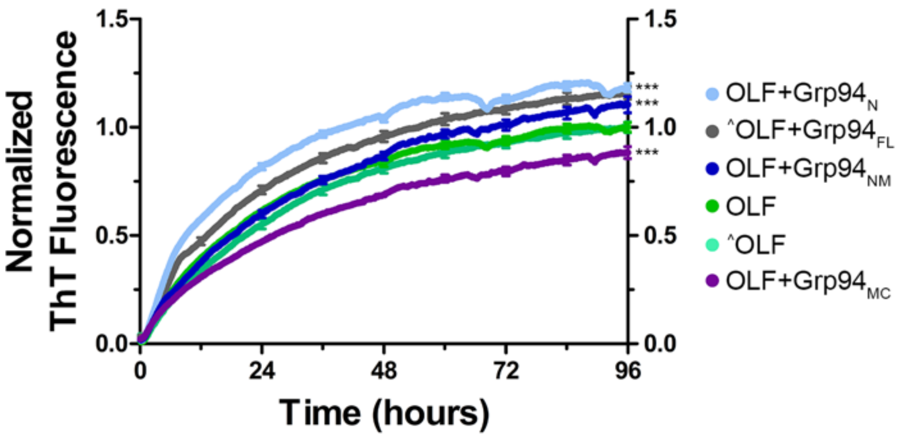


**Figure S1. Late-stage OLF aggregation kinetics continue early-data trends revealing that the N-terminal domain of Grp94 facilitates myocilin aggregation.** Effects of Grp94 domain constructs on OLF aggregation kinetics. Data correspond to kinetics time-course in Figure 2A, through t = 96 hours. The ^ symbol indicates the presented data were previously published[23]; *** ($p < 0.0001$) represents statistically significant differences relative to OLF.

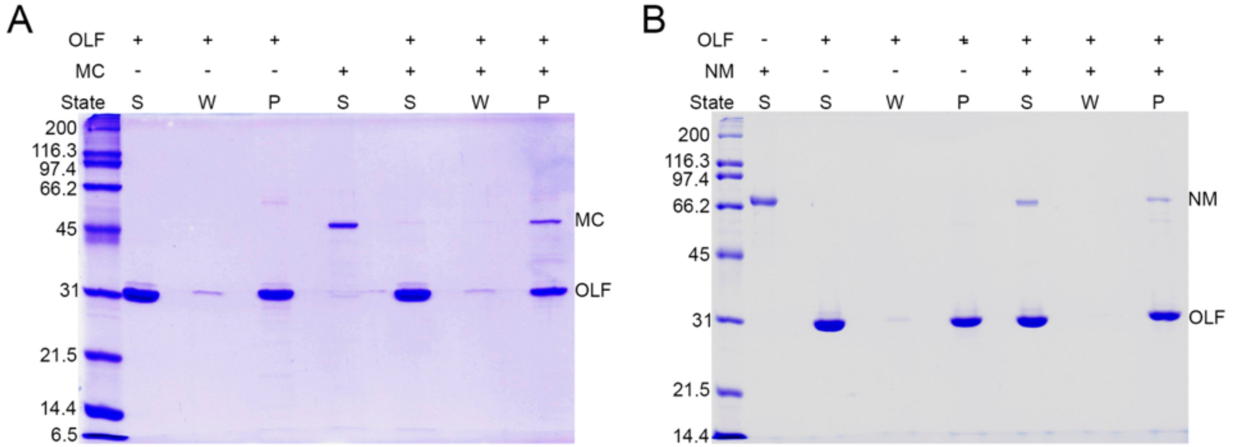


**Figure S2. The M domain of Grp94 stabilizes OLF, partially protecting it from aggregation.** SDS-PAGE analysis end-of-assay aggregates (Figure 2A) reveals extent of co-aggregation of OLF with (A) $Grp94_{MC}$ and (B) $Grp94_{NM}$. S = supernatant, W = wash, and P = pellet/aggregate. Densitometric quantification of OLF bands is presented in Table 1.

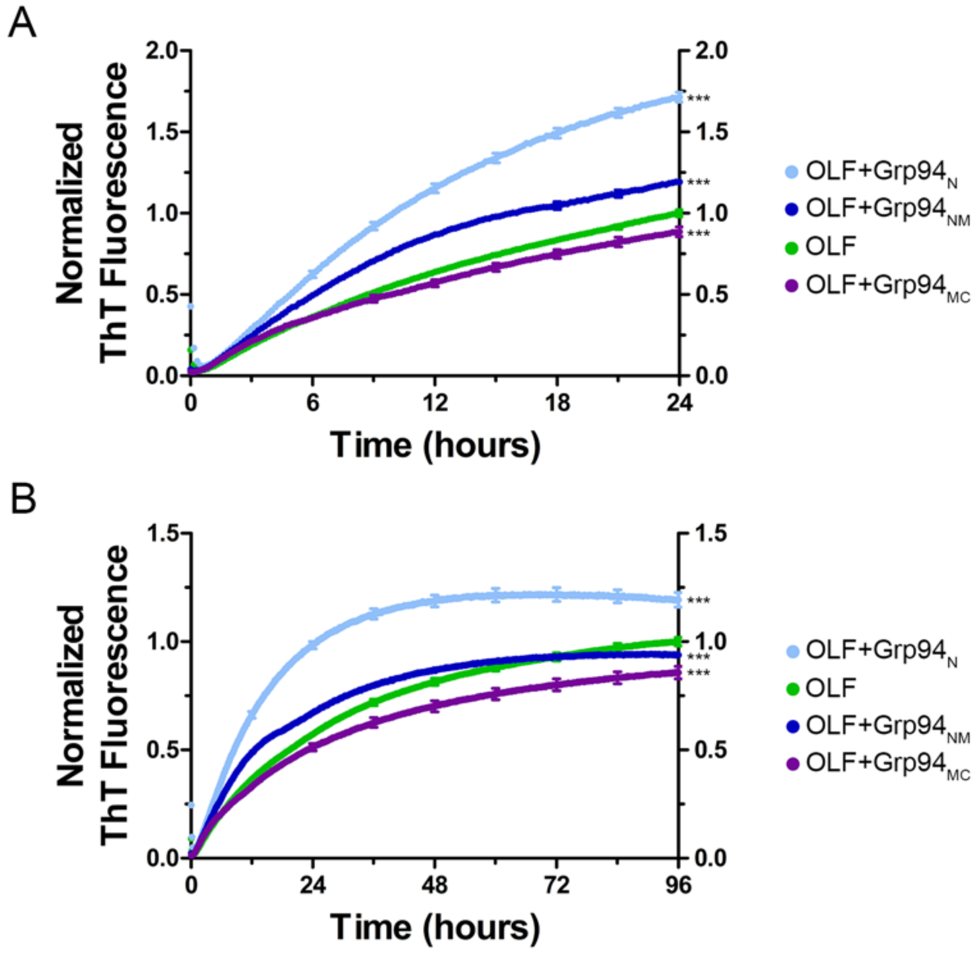


**Figure S3. Trends in OLF aggregation kinetics consistent with Grp94 and OLF proteins at stoichiometric levels**. Similar to the substoichiometric experiments (1:10 Grp94 domain:OLF, Figures 2A, S1), at elevated concentrations (1:1 ratio) $Grp94_N$ enhances OLF aggregation while $Grp94_{MC}$ stabilizes OLF as visualized in early- (A) and late-stage (B) kinetics traces. Results represent the average of 12 ($Grp94_N$), 12 ($Grp94_{NM}$), and 13 ($Grp94_{MC}$) replicates over 2 biological replicates; *** ($p < 0.0001$) indicates statistically significant differences relative to OLF at either 24 hours (A) or 96 hours (B).

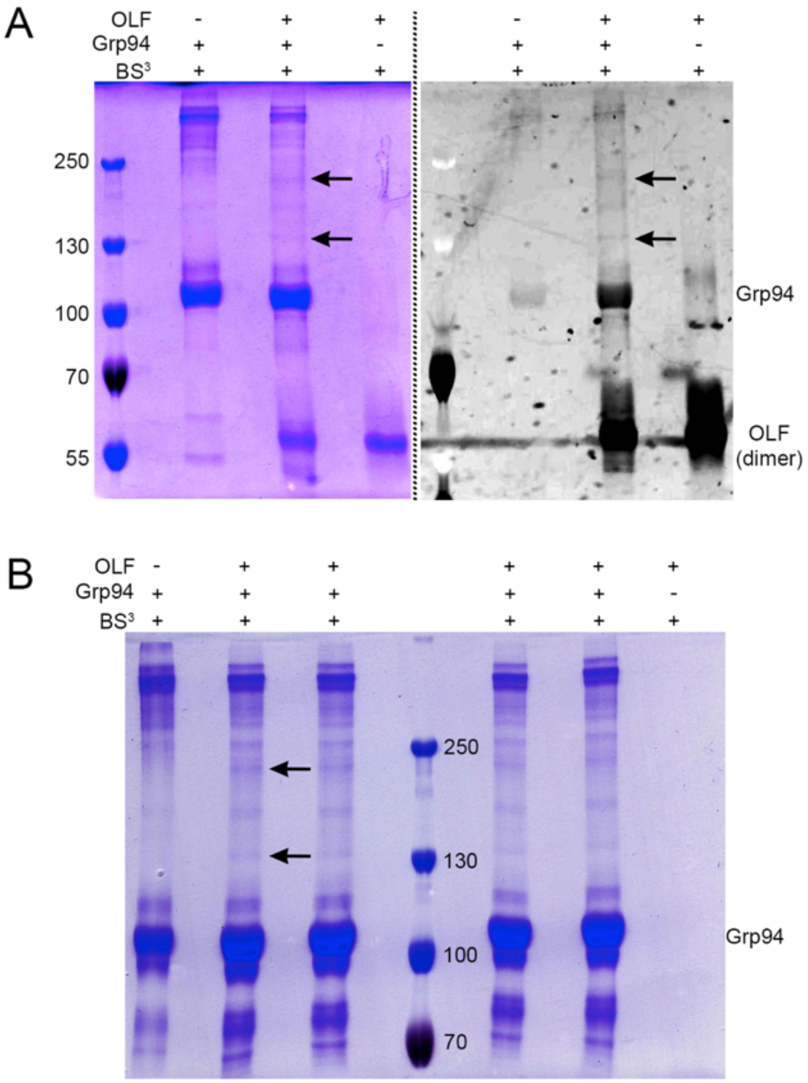


**Figure S4. Chemical crosslinking of full-length Grp94 with OLF.** Near-stoichiometric amounts of $BS^3$ were used to crosslink $Grp94_{FL}$ and OLF under OLF-destabilizing conditions. A) SDS-PAGE analysis of $Grp94_{FL}/OLF_d$ crosslinked products showing sparse hetero-species products as visualized by Coomassie blue staining (panel left of dashed line) and dansyl fluorescence (panel right of dashed line). See Figure S11A for uncropped gel images. B) Scaled-up crosslinking reaction product gel utilized for mass spectrometry analysis. Arrows indicate hetero-species products.

**Table S1. Anticipated masses of Grp94/OLF crosslinked species.**

| Protein Species | Expected Mass (kDa) |
|---|---|
| OLF | 31.3 |
| OLF···OLF | 62.5 |
| Grp94 | 93.9 |
| Grp94···Grp94 | 187.7 |
| Grp94···OLF | 125.1 |
| Grp94···OLF···Grp94 | 219.0 |
| N | 36.0 |
| N···OLF | 67.2 |
| NM | 69.8 |
| NM···OLF | 101.1 |
| MC | 51.6 |
| MC···MC | 103.2 |
| MC···OLF | 82.9 |
| MC···OLF···MC | 134.5 |

Crosslinking indicated by ··· .

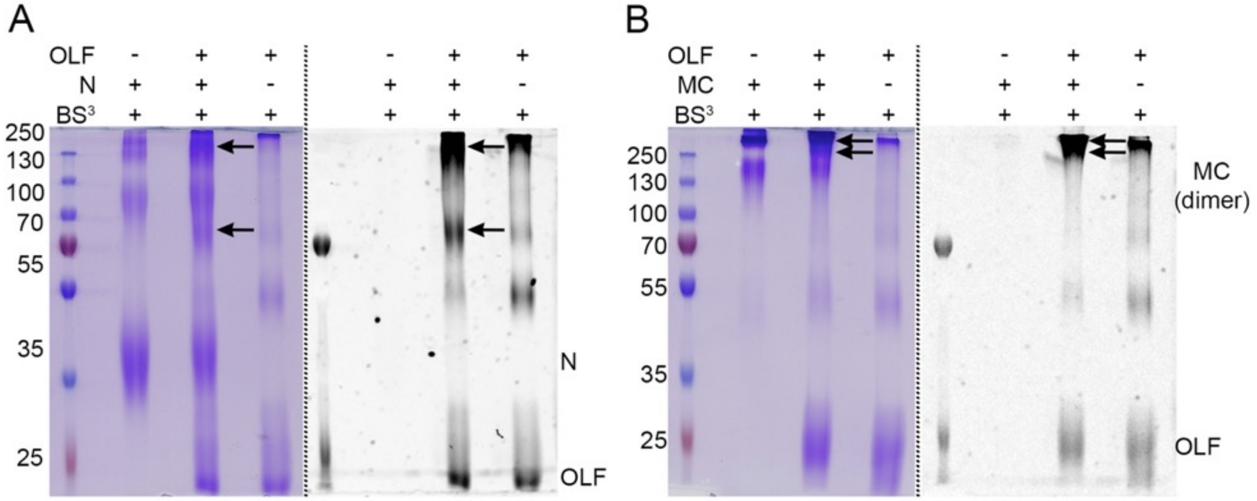


**Figure S5. Chemical crosslinking reactions with excess $BS^3$ results in the formation of larger quantities of less-discrete hetero-species products**. Coomassie blue (panels left of dashed lines) and dansyl-fluorescence (panels right of dashed lines) visualization of SDS-PAGE analysis highlighting cross-linking reaction products for $Grp94_N$/OLF (A) and $Grp94_{MC}$/OLF (B) reactions. Arrows indicate the presence of captured hetero-species products. Reactions were repeated with unmodified OLF for downstream mass spectrometry analysis (Figure S6). See Figure S11C-D for uncropped gel images.

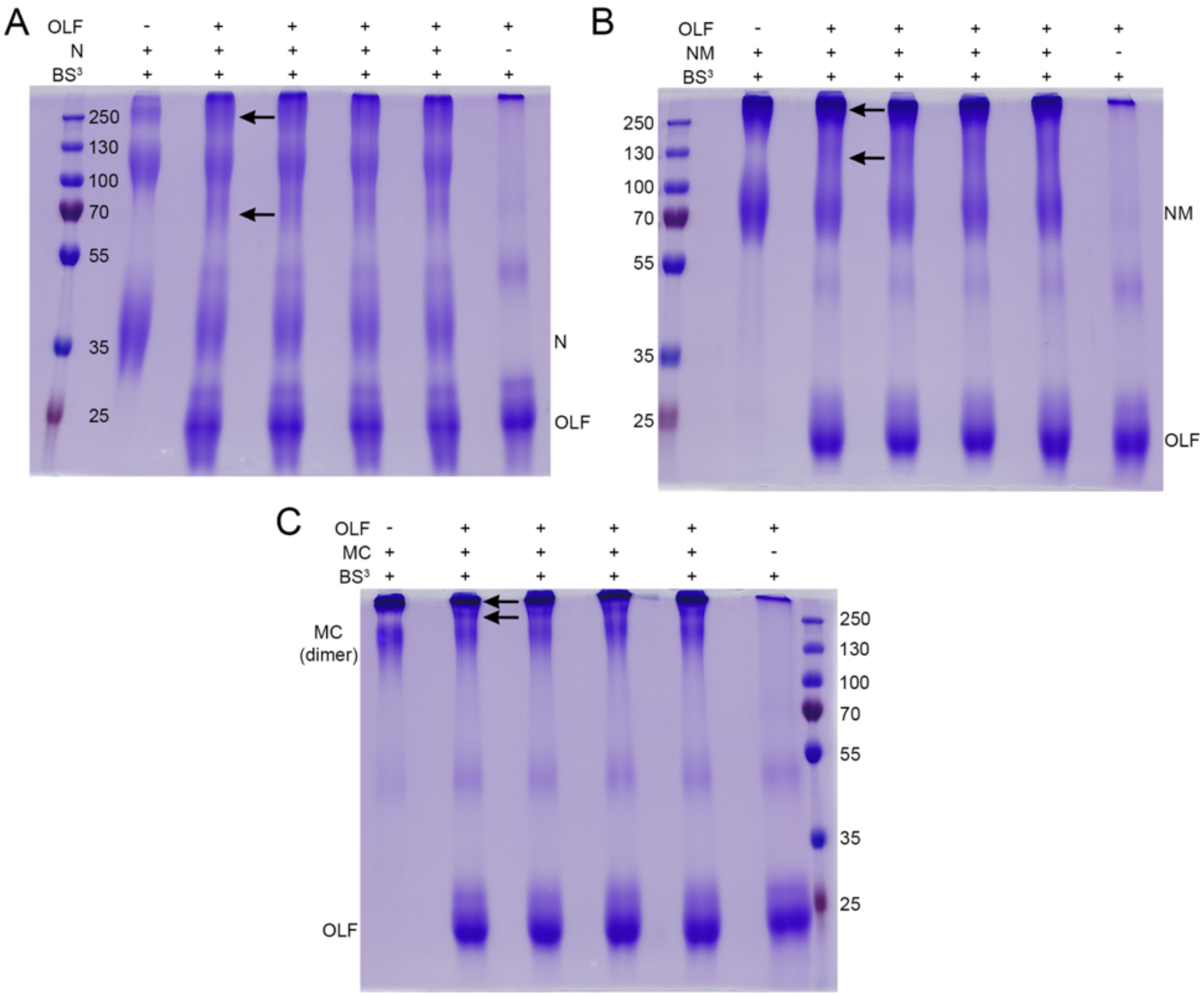


**Figure S6. Capture of Grp94 domain construct/OLF interaction complexes facilitated by crosslinking reactions employing excess $BS^3$.** Scaled-up reaction product SDS-PAGE analyses for reactions between (A) $Grp94_{N}$, (B) $Grp94_{NM}$, and (C) $Grp94_{MC}$ and OLF. Arrows indicate hetero-species products excised for mass spectrometry analysis.

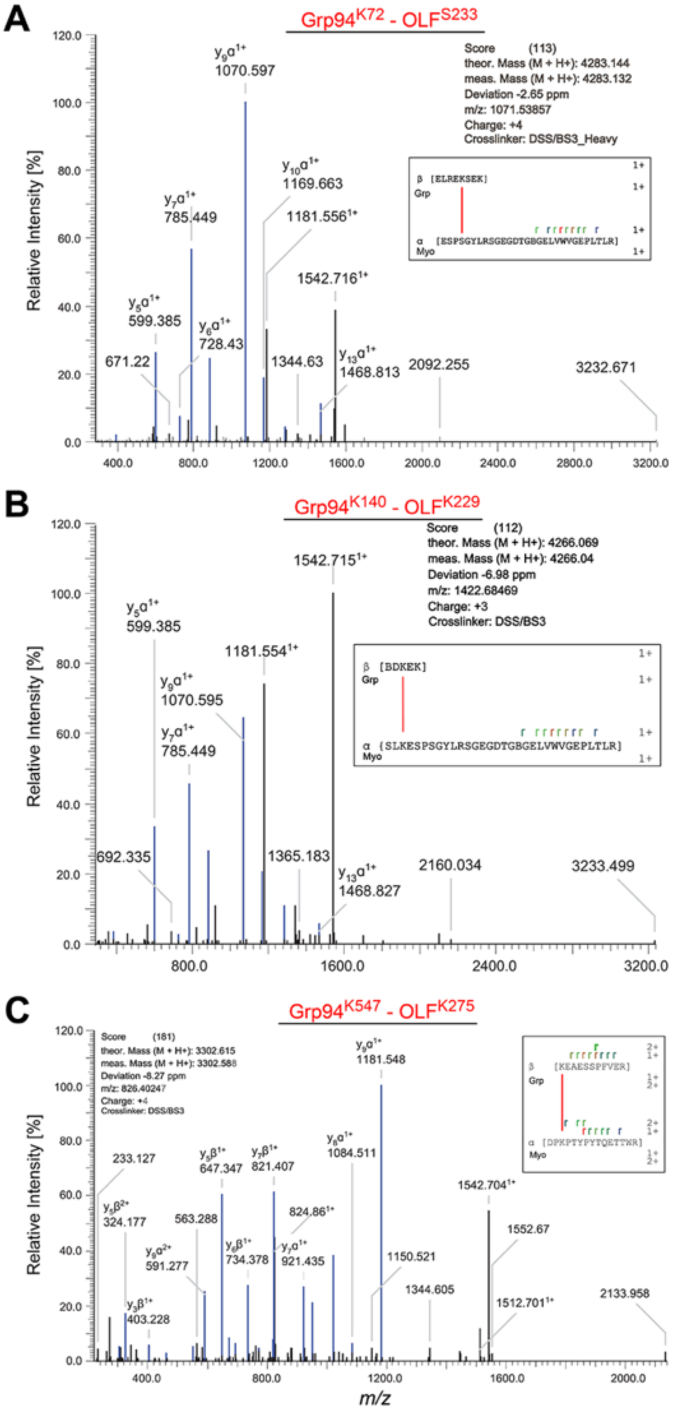


**Figure S7. Representative MS/MS spectra for lower frequency heteromeric crosslinks detected between Grp94 domains and OLF.** HCD fragmentation spectra with y-ion and b-ion assignments consistent with the crosslinked peptides shown (inset diagrams). A) Crosslink between Grp94$^{K72}$ and OLF$^{S233}$ (1 of 2 PSMs observed). B) Crosslink between Grp94$^{K140}$ and OLF$^{K229}$ (1 PSM). C) Crosslink observed between Grp94$^{K547}$ and OLF$^{K275}$ (1 PSM). Data correspond to gels presented in Figure S6A-B. Cross-linking sites are summarized in Table S2-tabs 2-3 and Figures 3C and 4. When the site of crosslinking was ambiguous, lysine sites of missed trypsin cleavage were indicated by default.

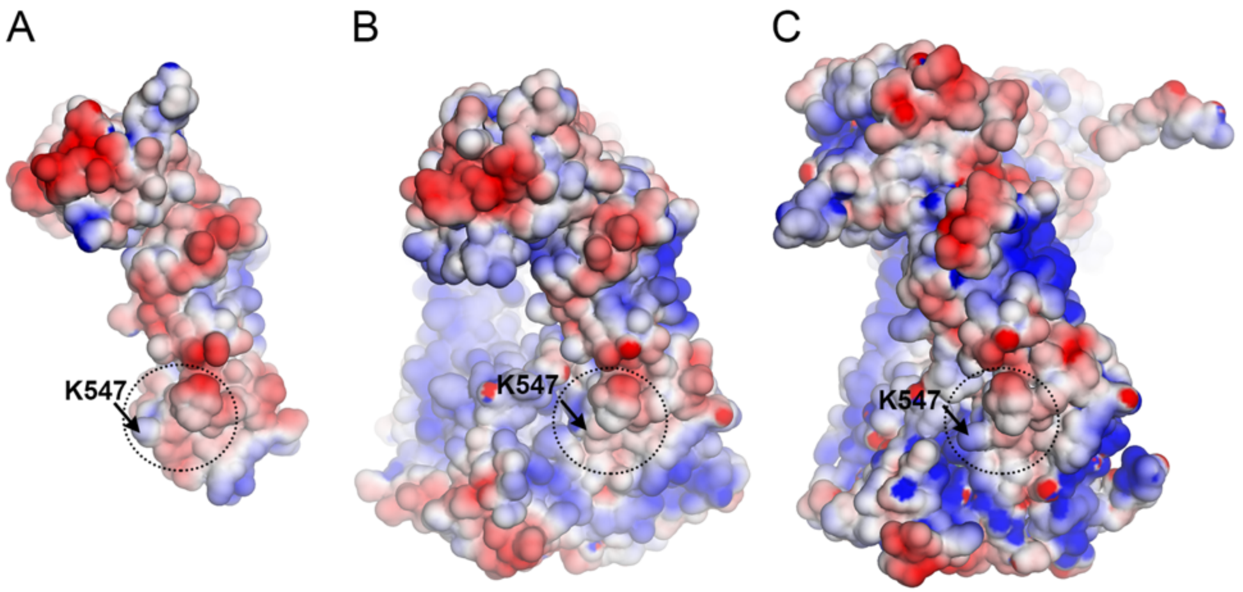


**Figure S8. Comparison of the electrostatic surface potential maps of available Grp94 structures in various conformations.** A) Surface potential modeled onto structure of $Grp94_{NM}$ (PDB-ID: 2O1W). The detected M crosslink site is exposed in a largely negatively-charged surface patch. B) Electrostatic surface potential of "partially closed" chaperone conformation ($Grp94_{FL}$ ADP-bound structure, PDB-ID: 2O1V). This model lacks the C-terminal-most 54 residues, which are primarily acidic, but conformationally overlays well with the MC model (Figure 4A, rmsd 2.3 Å over 826 residues). The M domain crosslinking site is solvent accessible. C) Surface potential modeled onto "fully closed" $Grp94_{FL}$ model (PDB-ID: 5ULS). This model, while lacking the 55 C-terminal residues of the C-domain, illustrates that the M crosslink site is surface-available. The electrostatic surface potentials, calculated with the PDB2PQR server (see Experimental Procedures), are colored negative (red, -5 kT/$e^-$) to positive (blue, 5 kT/$e^-$). The M domain crosslinking site is indicated by arrows.

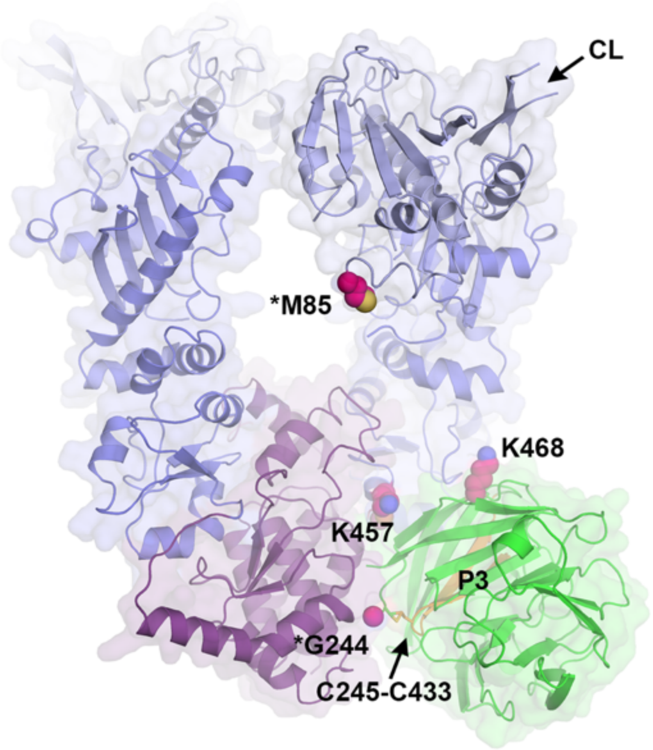


**Figure S9. Model of docking interaction between Grp94 and OLF.** Side-on interaction of OLF β-propeller with crosslink $Grp94^{K457}$-$OLF^{K468}$ used as a restraint. This model does not exclude the possibility of the far N region of Grp94 recognizing the OLF-proximal linker of myocilin. Structural model prepared with pyDockWEB server as outlined in Experimental Procedures, using structure files with PDB-ID codes 2O1V (ADP-bound $Grp94_{FL}$) and 4WXQ (OLF). Color scheme as in Figure 1, with key residues shown as magenta spheres. CL = charged linker, P3 = $V_{426}\cdots Y_{442}$ and is colored orange. The * indicates the presence of more, unstructured/absent N-terminal residues.

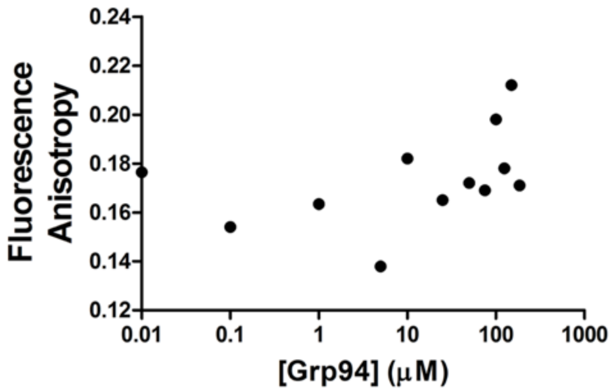


**Figure S10. Fluorescence polarization fails to detect binding between Grp94 and disease-related OLF(D380A).** Binding of the molecular chaperone to $OLF_d$(D380A) was undetectable over the concentration range tested, up to ~20,000:1 Grp94(monomer):OLF.

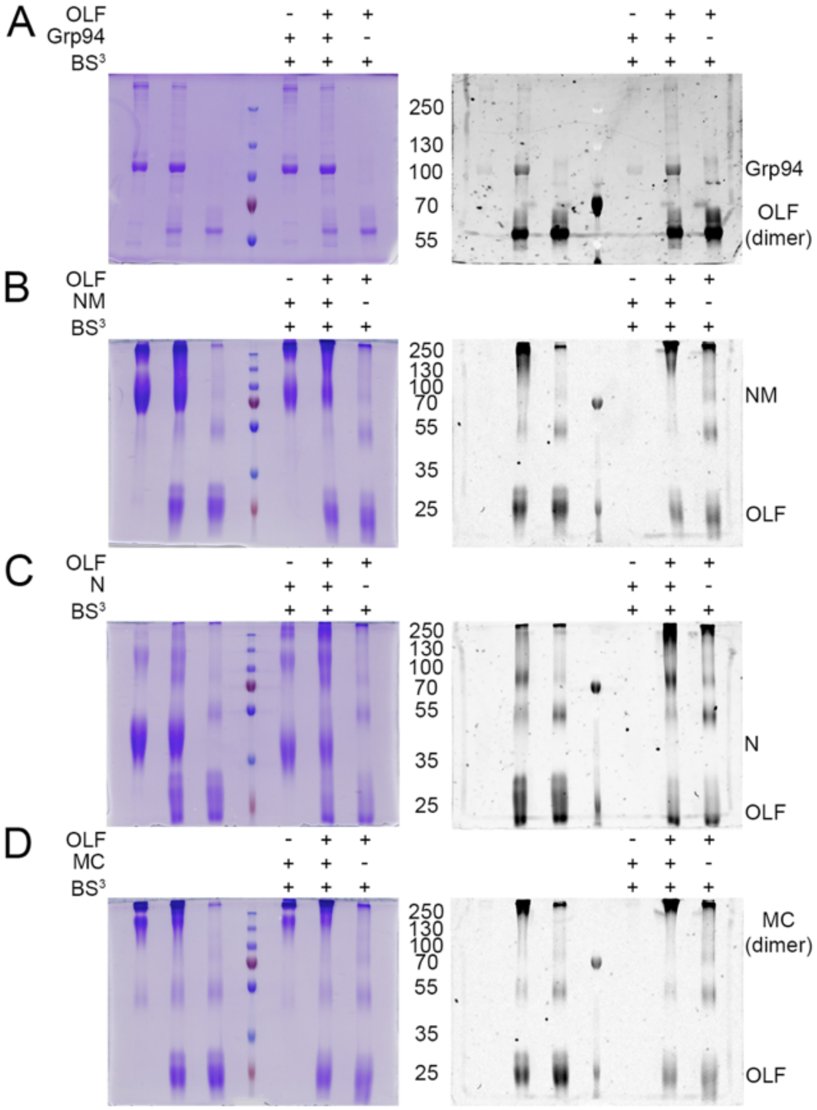


**Figure S11. Visualization by SDS-PAGE of $BS^3$ crosslinking products between Grp94/domains and OLF.** Uncropped gel images presented in (A) Figure S4A, (B) Figure 3A, (C) Figure S5A and (D) Figure S5B.

**Table S3. Table of primers utilized for site-directed mutagenesis to prepare Grp94$_N$, Grp94$_{NM}$ constructs.**

| Construct Prepared | Grp94 Variant | Primer Sequence (5′-3′) |
|---|---|---|
| N | D338Stop | CTGGGACTGGGAACTTATGAATTAGATCAAACCAATATGGCAGAG |
| | | CTCTGCCATATTGGTTTGATCTAATTCATAAGTTCCCAGTCCCAG |
| NM | G595Stop | CCAGAATGTTGCCAAGGAATGAGTGAAGTTCGATGAAAG |
| | | CTTTCATCGAACTTCACTCATTCCTTGGCAACATTCTGG |
